# A 1000 cc Intragastric Balloon for Weight Reduction

**DOI:** 10.1155/DTE.5.31

**Published:** 1998

**Authors:** A. Lavy, J. Lachter, O. Zinder, S. Eidelman

**Affiliations:** 1 Gastroenterology Unit Rambam Medical Center Haifa Israel; 2 Department of Laboratory Medicine Rambam Medical Center Haifa Israel

## Abstract

*Objective*: To examine the efficiency of a 1000 cc intragastric balloon as an aid for weight reduction.

*Subject*: Thirty-three morbidly obese persons, after multiple attempts for weight loss.

*Design*: Intragastric 1000 cc balloons were endoscopically inserted into the stomachs of subjects. The balloons were left in place for six months. During that period the patients received dietary therapy. Hormone levels were measured prior to insertion and upon withdrawal of balloons.

*Results*: After a brief initial weight loss, most patients regained the weight initially lost. Mean weight loss after six months was only 8 kg (range 0–22 kg). We found that Bombesin levels, but not Gastrin levels, were significantly lower at the end of therapy than before insertion of the balloon.

*Conclusion*: We conclude that a large intragastric balloon for a prolonged time did not assist in weight loss. Our preliminary results show Bombesin levels change significantly, a finding which warrants further study.


*Diagnostic and Therapeutic Endoscopy*, Vol. 5, pp. 31–35
Reprints available directly from the publisher
Photocopying permitted by license only

© 1998 OPA (Overseas Publishers Association) N.V.
Published by license under
the Harwood Academic Publishers imprint,
part of The Gordon and Breach Publishing Group.
Printed in Singapore.

### A 1000 cc Intragastric Balloon for Weight Reduction

A. LAVY[a,*], J. LACHTER[a], O. ZINDER[b] and S. EIDELMAN[a]

[a]*Gastroenterology Unit,* [b]*Department of Laboratory Medicine, Rambam Medical Center, Haifa, Israel*

*(Received 22 December 1997; In final form 30 March 1998)*

***Objective***: **To examine the efficiency of a 1000 cc intragastric balloon as an aid for weight reduction.**
***Subjects***: **Thirty-three morbidly obese persons, after multiple attempts for weight loss.**
***Design***: **Intragastric 1000 cc balloons were endoscopically inserted into the stomachs of subjects. The balloons were left in place for six months. During that period the patients received dietary therapy. Hormone levels were measured prior to insertion and upon withdrawal of balloons.**
***Results***: **After a brief initial weight loss, most patients regained the weight initially lost. Mean weight loss after six months was only 8 kg (range 0–22 kg). We found that Bombesin levels, but not Gastrin levels, were significantly lower at the end of therapy than before insertion of the balloon.**
***Conclusion***: **We conclude that a large intragastric balloon for a prolonged time did not assist in weight loss. Our preliminary results show Bombesin levels change significantly, a finding which warrants further study.**

*Keywords:* Intragastric balloon, Obesity, Bombesin, Gastrin

### INTRODUCTION

Use of intragastric balloons as part of the therapeutic intervention program for weight reduction is an attractive theoretical approach that has met with technical difficulties. This caused most physicians to currently forego this option in treating obesity [1]. Over the past decade, several types of intragastric balloons were tested and not found to be definitely better than other nonsurgical approaches [2,3]. The Garren–Edwards balloon, a 200 cc bubble, was found to be associated with serious complications [4]. The 500 cc Danish Balloobes Bubble was found to be safe but not more effective than a very low calorie diet with medical and dietary support [5,6]. The purpose of the present study was to test the hypotheses that a larger volume balloon and/or a longer duration of insertion would be more effective than those previously used, without compromising patient safety. An additional objective of this research was to study the effect of gastric distension on serum Bombesin and Gastrin levels.

* Corresponding author.

### METHODS

Thirty-three morbidly obese patients were admitted to an open trial. Criteria for inclusion in the study were severe obesity, failure at maintaining weight loss from previously attempted non-invasive approaches, and fully informed consent as required by the Helsinki Committee. Severe obesity was defined using the National Health and Nutrition Examination Survey (NHANESII) criteria [7]. Exclusion criteria were the presence of severe systemic disease, prior gastric surgery, peptic ulcer disease, primary endocrine disorders, coagulation disturbances, age under 18 years, and pregnancy. The patients included nine men, mean age 39 years (range 18–46 years), and 24 women, mean age 36 years (range 21–66). The mean weight for men was 108.7 kg (range 100–140 kg); for women mean weight was 108.4 kg (range 95–140 kg).

Blood count, coagulation evaluation, and serum electrolytes were examined prior to insertion, and following removal of the balloon.

### Dietary Assessment

Every patient was closely followed with twice-monthly meetings with a dietician, and weekly physician follow-up, but did not receive behavioral modification therapy. The dietician took extensive dietary histories, created a personalized diet plan for every patient, allowing for 1000–1200 calories daily, and including a multivitamin supplement. Follow-up dietician visits focused on allowable substitutions within the caloric framework, and reinforcement.

### Balloon Qualities

The balloon used was produced by the Tremco Company (Nethanya, Israel) from a mixture of synthetic thermostatic elastomeres, enabling the 1000 cc volume expansion without loss of stability. It had a smooth surface with radio-opaque surface markers. The procedure was performed on an outpatient basis. Following esophagogastroduodenoscopy the balloon was inserted alongside the endoscope, using a snare device to hold the balloon in place. After inflation of 1000 cc of air, the inflation catheter and endoscope were removed. Removal of balloons was performed by puncturing the balloon with a sclerotherapy needle, and, after deflation, snare removal. Volume stability and positioning was followed by plain film X-rays of the abdomen (see Fig. 1).

### Hormonal Measurements

There are several reports which have explored the associations and putative roles of peptide hormones and satiety [8]. The best studied are Cholecystokinin, Pancreatic Glucagon and Bombesin. Gastric dilatation causes gastric secretion in the rat, through activation of Bombesin neurons [9]. In the human, variable effects have been reported [10–12]. The influence of Bombesin on Gastrin release has been demonstrated in various situations [13]. We attempted to determine whether chronic gastric distension as caused by a

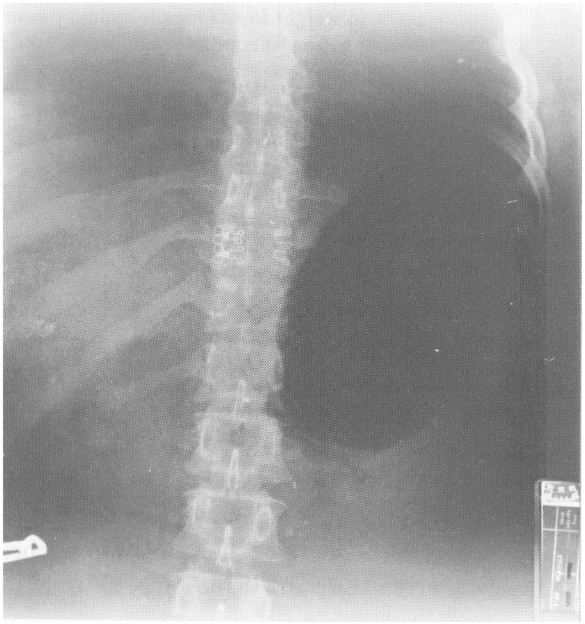

FIGURE 1 1000 cc air-filled balloon in the stomach.

balloon, would have any effect on Gastrin and/or Bombesin. Serum Bombesin and Gastrin levels were measured prior to insertion of the balloon, and six months after insertion of the balloon, before its withdrawal. Gastrin was measured by radioimmunoassay (CIS bioindustries GASK-PR kit, France). Bombesin was measured by radioassay using INCSTAR Bombesin 125/RIA kit MN, USA.

### RESULTS

Following insertion of the balloon, most patients experienced nausea, cramps, and vomiting lasting 3–5 days and all patients lost weight during the first three weeks. Over the next six months, only one patient complained of abdominal pain, occurring three months after insertion of the balloon. Endoscopy was performed and revealed a shallow gastric ulcer. The balloon was removed and the patient recovered. No other patient had notable side effects. There were no extraction difficulties in removing the balloons.

After the short-lived initial weight loss most patients ceased to lose weight and then proceeded to regain the weight initially lost (see Table I and Fig. 2). Mean weight lost after six months was 6 kg (range 0–22 kg). A frequent sensation experienced by patients was abdominal discomfort relieved by eating, and it was thus difficult for them to skip or reduce the size of meals.

The results of the peptide hormonal studies were as follows: prior to insertion of the balloon Gastrin level (pg/ml) was 46.3 SD 2.8. After six months, prior to withdrawal, the Gastrin level was 41.2 SD 2.2. This difference showed a nonsignificant trend towards decreased gastrin distension. Bombesin level prior to insertion was 39.4 SD 5.9 pg/ml, and six months later, before balloon withdrawal was 16.9 SD 3.4 pg/ml. This difference was statistically significant, $p < 0.001$ (see Table II).

We found no correlation between Bombesin level and the weight of the patients.

TABLE I Table of sex, age and weight of patients

| Age | Weight (in kg) | | | |
|---|---|---|---|---|
| | Initial | At 2 weeks | At 6 months | Change |
| Females | | | | |
| 37 | 110 | 100 | 110 | 0 |
| 44 | 95 | 87 | 96 | 1 |
| 41 | 109 | 101 | 109 | 0 |
| 36 | 100 | 94 | 100 | 0 |
| 46 | 115 | 107 | 103 | 12 |
| 21 | 130 | 122 | 118 | 12 |
| 32 | 95 | 79 | 95 | 0 |
| 33 | 133 | 129 | 133 | 0 |
| 30 | 100 | 96 | 100 | 0 |
| 28 | 130 | 122 | 121 | 9 |
| 30 | 96 | 93 | 74 | 22 |
| 30 | 97 | 90 | 91 | 6 |
| 38 | 105 | 101 | 105 | 0 |
| 66 | 133 | 127 | 128 | 5 |
| 45 | 96 | 90 | 84 | 12 |
| 45 | 100 | 95 | 87 | 13 |
| 44 | 111 | 109 | 111 | 0 |
| 21 | 120 | 120 | 120 | 0 |
| 21 | 130 | 130 | 130 | 0 |
| 29 | 106 | 100 | 106 | 0 |
| 32 | 101 | 96 | 84 | 17 |
| 43 | 98 | 92 | 88 | 10 |
| 37 | 95 | 89 | 85 | 10 |
| 29 | 97 | 93 | 97 | 0 |
| Average | | | | |
| 36 | 108.4 | 102.6 | 103.1 | |
| Males | | | | |
| 46 | 109 | 103 | 109 | 0 |
| 45 | 102 | 97 | 88 | 14 |
| 39 | 108 | 102 | 103 | 5 |
| 40 | 100 | 92 | 81 | 19 |
| 42 | 113 | 106 | 98 | 15 |
| 18 | 105 | 98 | 85 | 20 |
| 44 | 100 | 90 | 100 | 0 |
| 45 | 102 | 96 | 103 | −1 |
| 37 | 140 | 138 | 140 | 0 |
| Average | | | | |
| 39 | 108.7 | 102.4 | 100.7 | |

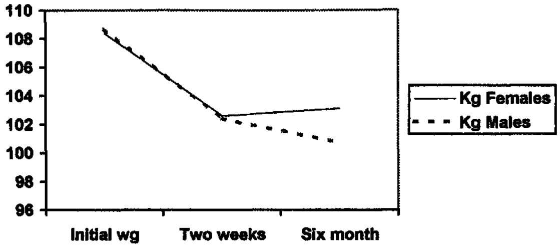

FIGURE 2 Mean weight loss for men and women.

TABLE II Mean Gastrin and Bombesin serum levels before and six months after balloon insertion

| | Gastrin (pg/ml) | Bombesin (pg/ml) |
|---|---|---|
| Prior to insertion | 46.3 (SE = 2.8) | 39.4 (SE = 5.9) |
| 10 min after insertion | 48.6 (SE = 2.8) | |
| Prior to withdrawal | 41.2 (SE = 2.2) | 16.9 (SE = 3.4)* |
| 10 min after removal | 41.4 (SE = 1.9) | |

SE = Standard error.
*$p < 0.001$ (Student's $t$-test).

### DISCUSSION

Mathus-Vliegen and Tytgat [5] in 1990 asked the question: "Would a higher-volume and longer-lasting balloon promote a greater weight loss or prolong adherence and compliance to treatment?" The present study was designed specifically to answer this important question. The balloon was designed to allow for a 1000 cc volume and was left in place for six months, as compared to recommended usage of the Garren–Edwards balloon, using 200 cc for 12 weeks. The balloon was unique in being air-filled rather than fluid-filled. Because our focus was to examine the result of a high volume balloon, we chose to use a constant volume of air, rather then adjust volume to the patient, as had been suggested at the Tarpon Springs Comprehensive Workshop [4]. Our balloon did not produce significant long-term weight loss. Our patients came to twice-monthly dietician and weekly physician interviews, but continued to experience hunger and discomfort which was relieved by eating. We could not establish whether this discomfort and cramping were the result of the balloon, a possible side effect, or from hunger related to the eating habits of the patients. Patients indicated that their hunger returned to the level it had been at before the balloon insertion, only 2–3 weeks after the balloon was inserted. Dietary assessments by physicians and dieticians, including use of substitutions and considerable encouragement and support, aiming for 1000–1200 kcal/day diets, left us with the impression that patients eating habits were unaffected by the balloon after the initial period of adaptation to its effect.

We found the level of Bombesin but not of Gastrin to be significantly reduced after the six months of gastric distension by the balloon. Short-term studies of gastric dilatation have shown that the level of distension is correlated with changes in Gastrin levels in the rat. Perhaps because of a lack of impressive weight loss by the end of the six month duration of the study, our data did not establish a significant relationship between the level of weight loss and the level of change of Gastrin or Bombesin when matched per individual, but the mean level of Bombesin for the group was significantly reduced. Why did Bombesin levels drop significantly in patients who had adapted to an intragastric balloon? Bombesin has been described as the hormone most likely to be responsible for satiety. Unlike Gastrin and Somatostatin, Bombesin retains its satiety effect even after complete denervation of the stomach [14]. We speculate that perhaps adaptive mechanisms led to overriding of a satiety effect of reduced Bombesin.

In the National Institutes of Health Consensus Development Conference Statement, specific needs were identified in the research for more effective alternate forms of weight reduction therapy [15]. Until the physiology of morbid obesity is better understood and appropriate interventions developed, our efforts may best be directed towards elucidating the pathophysiology of obesity, and meanwhile towards behavior modification and other noninvasive and invasive approaches. One survey of 50 experts significantly viewed the potential for anorectic drugs in the next ten years as considerably better than at present [16].

Our patients did not experience weight loss for a significant duration. These patients were self selected; they were highly motivated, severely obese patients who had all failed in many previous attempts to lose weight by other means.

### CONCLUSIONS

We conclude that neither intragastric balloon size nor the duration of use of the balloon could be demonstrated to be critical factors in determining the effectiveness of a weight loss program utilizing this modality. The reduction in the serum level of Bombesin, but not of serum Gastrin, following six months of balloon distension suggest that these hormones may be involved in a new homeostasis which may prevent feeling satiety despite having a small available gastric volume. This may be the cause for failure of various trials aimed at reducing intake by causing early satiety.

### *Acknowledgements*

The authors gratefully acknowledge the technical assistance of Tremco Factory. No financial support was provided for this study.

### *References*

[1] Frank, B.B., Stern, W.R. and Fisher, A.H., eds. *Survey of Gastric Bubble Usage in the United States*. Rockville, MD: Fisher, 1987.
[2] Schapiro, M., Benjamin, S., Blackburn, G., Frank, B., Heber, D., Kozarek, R., Randall, R. and Stern, W. Obesity and the gastric balloon: A comprehensive workshop. *Gastrointest. Endosc.* 1987; **33**: 323–327.
[3] McFarland, R.J., Grundy, A., Gazet, J.C. and Pickington, T.R.E. The gastric balloon: A novel idea proved ineffective. *Br. J. Surg.* 1987; **74**: 137–139.
[4] Chumley, D.L. Reported complications on the Garren–Edwards gastric bubble: Obesity and the gastric balloon, a comprehensive workshop, Tarpon Springs, Florida, Santa Ana., CA: American Edwards Laboratories, 1987.
[5] Mathus-Vliegen, E.M.H., Tytgat, G.N.J. and Veldhuyzen-Offermans, E.A.M.L. Intragastric balloon in the treatment of superb-morbid obesity: Double-blind sham-controlled crossover evaluation of a 500 ml balloon. *Gastroenterol.* 1990; **99**: 362–369.
[6] Borody, T.J. Complications and side effects of balloobes intragastric balloons in 427 patients. Balloobes International Symposium, Stockholm, 1988.
[7] National Center for Health Statistics. Plan and operation of the National Health and Nutrition Examination Survey, 1976–80. Washington, DC: US Public Health Service. PHS Publications no.(PHS) 81–1317, 1981.
[8] Smith, G.P. and Gibbs, J. Are gut peptides a new class of anorectic agents? *Am. J. Clin. Nutr.* 1992; **55**: 283s–285s.
[9] Schubert, M.L. and Makhlouf, G.B. Gastrin secretion induced by distention is mediated by gastric cholinergic and vasoactive intestinal peptide neurons in rats. *Gastroenterol.* 1993; **104**: 834–839.
[10] Schiller, L.R., Walsh, J.H. and Feldman, M. Distension induced gastrin release. *Gastroenterol.* 1980; **78**: 912–917.
[11] Sjovall, M., Berglund, B., Lindenstendt, G., Olbe, L. and Lundell, L. Inhibition of gastrin release induced by fundic distention. *Scand. J. Gastro.* 1991; **26**: 1231–1239.
[12] Gratzinger, U., Rahfeld, J.F. and Olbe, L. Is there an oxyntopyloric reflex for release of gastrin in men? *Gastroenterol.* 1977; **73**: 753–757.
[13] Graham, D.Y., Opekun, A., Lew, G.M., Klein, P.D. and Walsh, J.H. Helicobacter pylori associated gastrin release in duodenal ulcer patients. *Gastroenterol.* 1991; **100**: 1571–1575.
[14] Morley, J.E. Appetite regulation: The role of peptides and hormones. *J. Endocrinol. Invest.* 1989; **12**: 135–142.
[15] Gastrointestinal Surgery for Severe Obesity. NIH Consensus, Dev. Conf. Consens. Statement. *Am. J. Clin. Nutr.* 1992; **55**: 615s–619s.
[16] Bray, G.A., York, B. and DeLany, J. A survey of the opinions of obesity experts on the causes and treatment of obesity. *Am. J. Clin. Nutr.* 1992; **55**: 151s–154s.

